# Unsupervised Domain Adaptation Algorithm for Time Series Based on Adaptive Contrastive Learning

**DOI:** 10.3390/e28030272

**Published:** 2026-02-28

**Authors:** Huayong Liu, Peng Lin

**Affiliations:** School of Automation, Hangzhou Dianzi University, Hangzhou 310018, China

**Keywords:** unsupervised domain adaptation (UDA), deep learning (DL), contrastive learning (CL), time series classification

## Abstract

Time series data find extensive applications in finance, healthcare, and industrial monitoring domains. However, analytical models targeting such data are subject to notable constraints imposed by the rigid independent and identically distributed (IID) assumption and the high cost of data annotation. Unsupervised Domain Adaptation (UDA) offers an effective remedy for these challenges, and Contrastive Learning (CL) has been widely integrated into UDA frameworks, owing to its robust feature representation and clustering capabilities. Nonetheless, existing CL-based UDA methods suffer from two key limitations: (1) fixed data augmentation strategies result in imbalanced intensity—excessive augmentation erodes sample semantics, while insufficient augmentation induces model overfitting; (2) distribution alignment strategies neglect hard samples which are the core carriers of domain shift, causing their domain adaptation signals to be overshadowed by a large number of normal samples and thus degrading alignment accuracy. To address these drawbacks, this paper proposes a time-series UDA algorithm, termed Adaptive Contrastive Learning Domain Adaptation (ACLDA), which incorporates two key components: (1) an adaptive feature enhancement module that integrates adaptive sample augmentation and CL, enabling the model to capture high-quality transferable features; (2) sample-level adaptive weights, introduced on the basis of class-level alignment via supervised CL, to emphasize the value of hard samples. Comparative experiments on multiple time-series datasets demonstrate that our ACLDA outperforms state-of-the-art domain adaptation methods in terms of average accuracy, verifying its superiority and providing a more robust solution for cross-domain time series analysis.

## 1. Introduction

Time series data are widely prevalent in various fields, including financial markets, healthcare, industrial monitoring, and climate forecasting  [1,2,3,4]. In industrial scenarios, fault diagnosis of rotating machinery vibration time-series signals can avoid equipment downtime losses in advance [5]. In the medical field, anomaly detection of time-series signals such as electrocardiogram (ECG) and electroencephalogram (EEG) is an important means for the early diagnosis of cardiovascular diseases and epilepsy [6]. However, time-series analysis models in these scenarios generally rely on the strong assumption that the distribution of training data (source domain) is consistent with test data (target domain). When there are domain shift scenarios between the source domain and the target domain, such as working condition fluctuations, environmental interference, or differences in data collection conditions, the inter-domain distribution shift will lead to a sharp decline in the generalization ability of the model, or even complete failure [7,8]. Secondly, the time cost and monetary cost of data annotation, as well as the subjectivity of manual annotation, are also important factors that cannot be ignored [9,10,11]. Unsupervised domain adaptation (UDA) technology for time series does not require labeled data in the target domain. It achieves model transfer by reducing the difference in domain distribution, which well solves the problems of domain difference and difficulty in obtaining labeled data, and has become a research hotspot and difficulty in the field of time series analysis [12,13,14].

**Figure 1:**
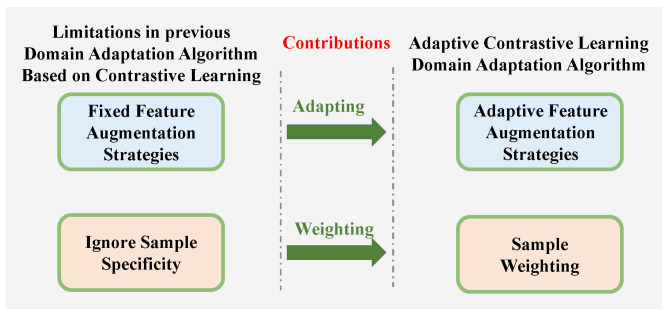
The contributions of ACLDA.

Contrastive learning (CL) has demonstrated strong capabilities in enhancing feature extraction for UDA by constructing positive and negative sample pairs [15]. However, despite its success, existing CL-based UDA approaches face two critical limitations shown in Figure 1. First, regarding feature enhancement, most methods rely on fixed data augmentation strategies [2,12,16,17]. This lack of adaptability often leads to either excessive augmentation (destroying semantic information) or insufficient augmentation (causing overfitting to surface features), both of which degrade generalization ability. Second, regarding distribution alignment, previous studies typically treat all samples with equal importance during the contrastive process [18,19,20,21]. This uniform weighting overlooks “hard samples”—data points that deviate from cluster centers and often carry the most significant domain shift information. If these hard samples are weighted equally with easy samples, the crucial domain adaptation signals they carry will be masked by the majority of normal features, preventing the model from capturing the core cues necessary for effective cross-domain alignment.

To address these challenges, this paper proposes a domain adaptation algorithm based on adaptive contrastive learning (ACLDA). The main contributions are summarized as follows:

- We propose an adaptive data augmentation strategy for feature enhancement. Unlike fixed methods, this approach dynamically adjusts augmentation intensity based on data characteristics. It effectively prevents semantic destruction caused by over-augmentation and superficial learning caused by under-augmentation, ensuring the extraction of robust transferable features.
- We introduce an adaptive weighted supervised contrastive learning loss. By automatically assigning higher weights to hard samples, this mechanism prevents critical domain-shift cues from being masked by easy samples. This significantly improves the model’s adaptability to complex cross-domain scenarios.
- Extensive experiments demonstrate the superiority of ACLDA. Through the synergistic effect of adaptive augmentation and weighted contrastive learning, our method achieves refined class-level distribution alignment, characterized by high intra-class compactness and clear inter-class separability.

The remaining parts of this paper are organized as follows: Section 2 reviews related work; Section 3 details the proposed ACLDA algorithm; Section 4 presents the experimental results; and Section 5 concludes the paper.

## 2. Related Work

### 2.1. Unsupervised Domain Adaptation

Unsupervised domain adaptation alleviates the distribution discrepancy between the source and target domains by transferring knowledge learned from the labeled source domain to the unlabeled target domain [22]. Maximum Mean Discrepancy (MMD) is a classic distribution-based domain adaptation method that performs marginal distribution alignment by minimizing the distribution difference of features from the source and target domains in the Reproducing Kernel Hilbert Space (RKHS) [23]. However, MMD only focuses on the first-order statistical information of the distribution and cannot fully characterize complex data distributions. Maximum Mean Covariance Discrepancy (MMCD) combines MMD with Maximum Classifier Discrepancy (MCD) to construct a maximum mean covariance discrepancy metric [24]. Compared with MMD, MMCD captures more distribution information. Nevertheless, the aforementioned methods ignore the problem of intra-class data compactness. Chen et al. corrected the intrinsic graph and introduced a penalty graph to reduce the inter-domain distribution difference while ensuring the compactness of intra-class data, making features more discriminative [25]. Ganin et al. implemented adversarial-based unsupervised domain adaptation by introducing a Gradient Reversal Layer (GRL) into the model [26]. However, this method only aligns marginal distribution features and ignores class-level distribution alignment. The model proposed by Jiao et al. consists of a shared feature extractor and two task-specific classifiers. Through the adversarial training game among the three, the model can learn fault features with both class separability and domain invariance to adapt to cross-domain diagnosis requirements [27]. On the basis of marginal distribution alignment, Kuang et al. introduced conditional distribution adversariality to form a two-layer adversarial transfer learning framework, enabling the learning of class-separable diagnostic knowledge under imbalanced data [28]. Domain adaptation based on feature reconstruction is a type of technology that achieves cross-domain transfer through data reconstruction constraints, but there is often a natural conflict between reconstruction accuracy and class discriminability. Xu et al. adopted an iterative strategy with two reconstruction matrices to cyclically reconstruct the data matrix and update the common subspace for invariant feature learning [29]. Guo et al. proposed a Reconstruction-based Domain Adaptive Transfer Network (RDATN) and introduced class-level weights and sample-level weights into the loss function to mitigate the negative impact of anomalous classes in partial transfer learning [30].

### 2.2. Contrastive Learning in UDA

Contrastive learning aims to learn discriminative representations by pulling positive pairs closer and pushing negative pairs apart [15]. In the context of UDA, it has been widely adopted to enhance feature transferability. However, current research faces challenges in two aspects: data augmentation strategies and sample weighting mechanisms.

Data augmentation is crucial for constructing contrastive views. Chen et al. verified that data augmentation compels models to discard superficial redundant features and focus on core semantic characteristics [15]. Li et al. proposed masking rectangular regions to simulate missing information, enhancing intra-domain feature separability [31]. Pan et al. utilized random augmentation for self-supervised contrastive learning to perceive domain-invariant features [32]. Despite these advances, most existing UDA methods rely on pre-defined, fixed augmentation strategies. For instance, Eldele et al. mixed source and target data in a fixed proportion [16]. Wu et al. sequentially adopted four fixed augmentation methods (e.g., jittering, scaling) to obtain different views [2,17]. Darban et al. injected fixed anomaly patterns into normal samples to generate negative pairs for triplet learning [12]. While effective in specific settings, fixed augmentation lacks flexibility: insufficient intensity fails to drive the model to learn essential characteristics, while excessive intensity destroys original semantic features. Since the model evolves during training, fixed strategies often lead to mismatched augmentation.

Beyond augmentation, how to effectively utilize samples for alignment is another key challenge. Standard contrastive learning treats all samples equally. To address domain shift, recent works have incorporated pseudo-labels or instance-level mixing. Pang et al. selected reliable pseudo-labels of target data to construct positive/negative pairs for supervised contrastive learning [18,19]. Yu et al. generated virtual target data by mixing source data with target styles for instance contrastive learning [20]. Wang et al. performed alignment between samples with target statistical features and original samples to retain class discriminability [21]. However, these methods generally assign uniform importance to all samples, ignoring the critical role of “hard samples”—those deviating from cluster centers. In cross-domain tasks, hard samples are often the core carriers of domain shift. If weighted equally with easy samples, the adaptation signals from hard samples are easily masked by the large volume of easy samples, limiting the model’s ability to achieve refined alignment in complex scenarios.

## 3. Proposed Method: ACLDA

**Figure 2:**
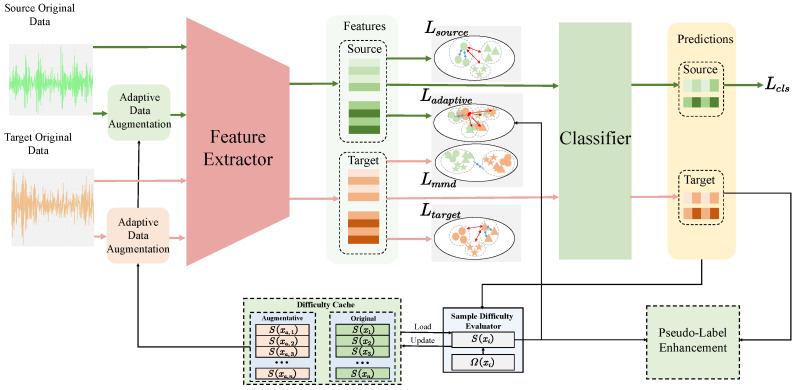
Overall architecture of ACLDA. the green arrows indicate the data flow direction of the source domain, while the red arrows denote the data flow direction of the target domain. In the feature space visualizations (e.g., $L_{source}$, $L_{target}$), different shapes (such as circles and triangles) represent different categories.

The proposed ACLDA framework aims to bridge the domain gap by learning higher-quality feature representations through adaptive contrastive learning. As depicted in Figure 2, the architecture consists of four key modules. The Sample Difficulty Perception module dynamically evaluates and caches the classification difficulty of samples. Based on these scores, the Feature Enhancement module applies adaptive data augmentation, ensuring that the feature extractor F(·) learns robust representations from samples. The Distribution Alignment Module achieves more precise distribution alignment through the synergy of MMD and weighted contrastive learning. The Pseudo-label Enhancement Module enhances the confidence of the prediction results for samples in the target domain.

The training workflow proceeds as follows: First, difficulty scores are retrieved to guide the generation of augmented views for both source and target batches. Then, these inputs are fed into the feature extractor to generate higher-quality feature representations via supervised contrastive learning on the source domain and self-supervised contrastive learning on the target domain. Crucially, we introduce a hybrid alignment strategy to align domain distributions. Global alignment is achieved via Maximum Mean Discrepancy (MMD). Meanwhile, weighted supervised contrastive learning is employed for fine-grained alignment, which leverages difficulty scores to prioritize hard samples during the alignment of source labels and target pseudo-labels. Finally, the classifier is optimized using source cross-entropy and target pseudo-label enhancement losses, with difficulty scores iteratively updated to reflect the model’s evolving state.

### 3.1. Sample Difficulty Perception

Mainstream difficulty-driven methods only use entropy as a direct metric for sample hardness, assuming that samples with higher entropy are more difficult to classify. Although such methods have been proven effective in other unsupervised or supervised classification scenarios, the model’s predictions on samples fluctuate significantly during training. If there are transient high-entropy samples, the model will treat them as hard samples, which will interfere with the model optimization direction. To address this issue, a sample difficulty evaluator is introduced to emphasize the impact of recent data while retaining long-term trends, thereby eliminating the interference of transient high-entropy samples on the training process.

#### 3.1.1. Instantaneous Classification Difficulty Evaluation

The sample difficulty evaluator first calculates the instantaneous classification difficulty score Ω(·) of a sample. For a source or target domain sample $x_{[a,]i}^{d}$ and its corresponding prediction $P_{[a,]i}^{d}$ (where $x_{a,i}^{d}$ denotes an augmented sample and $x_{i}^{d}$ denotes an original sample), the instantaneous classification difficulty score $\Omega (x_{[a,]i}^{d})$ is defined as follows:

(1) $$\begin{array}{c} \;\Omega \left(x_{[a,]i}^{d}\right)=\sqrt{\sum_{j=1}^{K}{\left(P_{[a,]i}^{d}\right)}_{j}^{2}\cdot \left(1-\delta_{j,z}\right)}\\ z=\left\{\begin{array}{c} k,\;d=s\\ \hat{k},\;d=t \end{array}\right.\;,\;\delta_{j,z}=\left\{\begin{array}{c} 1,\;j=z\\ 0,\;j\neq z\; \end{array}\right. \end{array}$$

where *K* denotes the number of classification categories, *k* represents the ground-truth label of the sample, and $\hat{k}$ denotes the pseudo-label of the sample (the pseudo-label is set to the category with the maximum prediction probability).

#### 3.1.2. Stable Classification Difficulty Evaluation

The instantaneous classification difficulty evaluation can only assess the sample classification difficulty in the current training iteration, which is prone to random fluctuations and thus lacks sufficient stability. The Exponential Moving Average (EMA) can effectively address this issue [33]. For a given instantaneous classification difficulty score $\Omega (x_{[a,]i}^{d})$, the method to calculate its stable classification difficulty score $S(x_{[a,]i}^{d})$ is as follows:

(2) $$\begin{array}{c} S\left(x_{[a,]i}^{d}\right)=\beta \cdot [S\left(x_{[a,]i}^{d}\right){]}^{\prime }+\left(1-\beta \right)\cdot \Omega \left(x_{[a,]i}^{d}\right) \end{array}$$

where $[S\left(x_{[a,]i}^{d}\right){]}^{\prime }$ denotes the stable classification difficulty score of the sample calculated in the previous mini-batch (retrieved from the Difficulty Cache). A larger weight β indicates greater emphasis on the previously calculated stable classification difficulty score of the sample, with less influence from the instantaneous classification difficulty score of the current sample.

#### 3.1.3. Difficulty Cache

The calculation of the stable classification difficulty score requires retrieving the stable classification difficulty score $[S\left(x_{[a,]i}^{s/t}\right){]}^{\prime }$ obtained from the previous mini-batch; thus, it is necessary to cache the stable classification difficulty score $S(x_{[a,]i}^{s/t})$ computed in each iteration. The stable classification difficulty score is also required in both the feature enhancement component and the distribution alignment component, but only the stable classification difficulty scores corresponding to augmented samples are used in the feature enhancement component. To facilitate the reading and writing of difficulty cache data, the difficulty cache is composed of two parts: the Augmentative Difficulty Cache (ADC) and the Original Difficulty Cache (ODC), which are responsible for storing the stable classification difficulty scores of original samples and augmented samples, respectively. For a sample $x_{i}$ with index idx, the stable classification difficulty score of the original sample can be retrieved from the ODC as $S\left(x_{i}\right)=\mathrm{ODC}\left[\mathrm{idx}\right]$, and the stable classification difficulty score of the augmented sample can be retrieved from the ADC as $S\left(x_{a,i}\right)=\mathrm{ADC}\left[\mathrm{idx}\right]$.

### 3.2. Feature Enhancement Module

To fully leverage the label information of the source domain and the unlabeled information of the target domain, while exploring the intrinsic value of samples through adaptive data augmentation to enhance the model’s robustness against domain discrepancies, this paper first dynamically generates adaptively augmented samples based on sample difficulty. Subsequently, supervised and self-supervised contrastive learning mechanisms are employed respectively according to the characteristics of the dual-domain data.

#### 3.2.1. Adaptive Data Augmentation

Traditional fixed data augmentation methods suffer from either insufficient augmentation or excessive distortion. Insufficient augmentation results in negligible differences between augmented samples and original samples, failing to provide the model with adequate effective perturbations. Excessive distortion causes augmented samples to lose the core semantic features of the original samples (e.g., fault types, structural patterns) and become meaningless noisy samples. To avoid the aforementioned issues, this paper adopts an adaptive data augmentation method. For a given sample $x_{i}^{s/t}$, its corresponding positively augmented sample via adaptive data augmentation $x_{\left[a,i\right]}^{s/t}$ is defined as follows:

(3) $$\begin{array}{c} x_{[a,]i}^{s/t}=\alpha \cdot T_{w}\left(x_{i}^{s/t}\right)+\left(1-\alpha \right)\cdot T_{s}\left(x_{i}^{s/t}\right) \end{array}$$

where $T_{w}$ denotes weak augmentation, such as small-scale time-step shifting and slight amplitude scaling; $T_{s}$ denotes strong augmentation, such as time stretching/compression, adding high-intensity mixed noise, and local signal replacement. α represents the stable classification difficulty score of the previous iteration $[S\left(x_{a,i}^{s/t}\right){]}^{\prime }$. A larger value of α indicates higher classification uncertainty of the augmented version of the sample, which leads to a larger proportion of weak augmentation and a smaller proportion of strong augmentation applied to the sample.

#### 3.2.2. Supervised Contrastive Learning for Source Domain

For the source domain features $F_{a}^{s}={\left\{f_{a,i}\right\}}_{i=1}^{n_{s}}$ and $F^{s}={\left\{f_{i}\right\}}_{i=1}^{n_{s}}$, since the source domain data are accompanied by labels, the labels corresponding to the augmented source domain samples are identical to those of the original samples. For the convenience of loss function representation, we define $F_{all}^{s}=\left\{F^{s},F_{a}^{s}\right\}={\left\{f_{i}\right\}}_{i=1}^{2n_{s}}$, and the corresponding supervised contrastive learning loss for the source domain is given as follows:

(4) $$\begin{array}{c} L_{s}=-\frac{1}{2n_{s}}\sum_{i=1}^{2n_{s}}\frac{1}{|P_{i}|}\sum_{p\in P_{i}}\log\frac{\exp(\mathrm{sim}\left(f_{i}^{s},f_{p}^{s}\right)/\tau )}{\sum_{q\in N_{i}}{\left(\frac{1}{N_{c_{q}}}\right)}^{\gamma }\exp\left(\mathrm{sim}\left(f_{i}^{s},f_{q}^{s}\right)/\tau \right)} \end{array}$$

where $sim(f_{i},f_{p})$ denotes the cosine similarity between features $f_{i}$ and $f_{p}$; τ represents a temperature parameter that adjusts the smoothness of similarity measurement, thereby affecting the sensitivity of the loss function to the similarity between data samples; $P_{i}$ denotes the positive sample set, which includes data belonging to the same fault type as feature $f_{i}$; $N_{i}$ denotes the negative sample set, which includes data belonging to different fault types from feature $f_{i}$; $N_{c_{q}}$ denotes the total number of samples in the class to which the negative sample *q* belongs; hyper-parameter γ modulate the intensity of class re-weighting.

#### 3.2.3. Self-Supervised Contrastive Learning for Target Domain

For the target domain features $F_{a}^{t}={\left\{f_{a,i}\right\}}_{i=1}^{n_{t}}$ and $F^{t}={\left\{f_{i}\right\}}_{i=1}^{n_{t}}$, since the fault types of the target domain data are unknown, self-supervised contrastive learning is adopted for the target domain data. An original sample and its corresponding augmented sample are mutual positive samples, while all other samples in the target domain are negative samples. The corresponding self-supervised contrastive learning loss for the target domain is given as follows:

(5) $$\begin{array}{c} L_{t}=-\frac{1}{n_{t}}\sum_{i=1}^{n_{t}}\log\frac{\exp(\mathrm{sim}\left(f_{i}^{t},f_{a,i}^{t}\right)/\tau )}{\sum_{q\in N_{i}}{\left(\frac{1}{N_{c_{q}}}\right)}^{\gamma }\exp\left(\mathrm{sim}\left(f_{i}^{t},f_{q}^{t}\right)/\tau \right)} \end{array}$$

where $N_{i}$ denotes all samples except the augmented sample corresponding to the target sample and the target sample itself; $N_{c_{q}}$ denotes the total number of samples in the class to which the negative sample *q* belongs; hyper-parameter γ modulate the intensity of class re-weighting.

#### 3.2.4. Feature Enhancement Loss

In the feature enhancement component, this paper adopts adaptive data augmentation to avoid the problems of insufficient augmentation and over-augmentation. Moreover, leveraging the data characteristics of different domains, contrastive learning is introduced to improve the quality of features learned by the model and enhance the model’s robustness against cross-domain discrepancies. The feature enhancement loss is denoted as $L_{\mathrm{aug}}$:

(6) $$\begin{array}{c} L_{aug}=L_{s}+L_{t} \end{array}$$

### 3.3. Distribution Alignment Module

The core challenge of unsupervised domain adaptation lies in the data distribution shift between the source domain and the target domain. Even though the representations are optimized through preliminary difficulty-aware adaptive augmentation and dual-domain contrastive learning, the source domain features may still retain domain-specific information, and the intra-class consistency and cross-domain compatibility of the target domain features have not yet been fully established. This paper uses the Maximum Mean Discrepancy (MMD) for global distribution alignment, optimizing the overall distribution distance between the source and target domains in the feature space to achieve macro-alignment of dual-domain features. Meanwhile, considering the importance of more refined distribution alignment strategies in the field of domain adaptation and the significance of hard samples for improving classification performance, the supervised contrastive loss is weighted to assign greater weights to hard-to-classify samples, thereby achieving sample-level class distribution alignment.

#### 3.3.1. MMD Distribution Alignment

The core idea of MMD is as follows: by mapping dual-domain samples to the Reproducing Kernel Hilbert Space (RKHS), the distance between the sample means of the two domains is calculated. The smaller this distance, the closer the distributions of the two domains are. For the source domain features $F_{all}^{s}=\left\{F^{s},F_{a}^{s}\right\}={\left\{f_{i}^{s}\right\}}_{i=1}^{2n_{s}}$ and target domain features $F_{all}^{t}=\left\{F^{t},F_{a}^{t}\right\}={\left\{f_{i}^{t}\right\}}_{i=1}^{2n_{t}}$, the MMD is defined as:

(7) $$\begin{array}{c} L_{mmd}={∥\frac{1}{2n_{s}}\sum_{i=1}^{2n_{s}}\phi \left(f_{i}^{s}\right)-\frac{1}{2n_{t}}\sum_{i=1}^{2n_{t}}\phi \left(f_{i}^{t}\right)∥}_{H}^{2} \end{array}$$

where ϕ(·) denotes the mapping from the feature space to the RKHS; H represents the Reproducing Kernel Hilbert Space, and ${∥\cdot ∥}_{H}$ denotes the norm of this space.

#### 3.3.2. Weighted Supervised Contrastive Learning

Supervised contrastive learning takes each sample as an anchor, regards data of the same fault type as positive samples, and data of different fault types as negative samples. This is equivalent to assuming that each sample has the same weight, which leads to the problem of easy samples dominating the training process and insufficient learning of hard samples. To address this issue, this paper adopts weighted supervised contrastive learning, assigning larger weights to hard samples and smaller weights to easy samples. The category of target domain data is determined by its corresponding prediction result $P_{all}^{t}$: the class with the maximum prediction probability is selected, provided that the probability of this class is greater than the threshold θ. For the source domain features $F_{all}^{s}=\left\{F^{s},F_{a}^{s}\right\}={\left\{f_{i}^{s}\right\}}_{i=1}^{2n_{s}}$ and target domain features $F_{all}^{t}=\left\{F^{t},F_{a}^{t}\right\}={\left\{f_{i}^{t}\right\}}_{i=1}^{2n_{t}}$, after filtering the target domain features according to the threshold, all features are merged and denoted as $F={\left\{f_{i}\right\}}_{i=1}^{n}$. The category of each feature is denoted as $Y={\left\{y_{i}\right\}}_{i=1}^{n}$, and the corresponding samples are denoted as $X={\left\{x_{i}\right\}}_{i=1}^{n}$. For a sample $x_{i}$, its corresponding weight is given as follows:

(8) $$\begin{array}{c} w_{i}=\frac{S(x_{i})}{\sum_{j=1}^{n}I\left(y_{i}=y_{j}\right)S\left(x_{j}\right)} \end{array}$$

where I(·) denotes the indicator function.

Once the weight of each sample is obtained, the weighted supervised contrastive learning loss $L_{w}$ can be formulated as follows:

(9) $$\begin{array}{c} L_{w}=-\frac{1}{n}\sum_{i=1}^{n}\frac{w_{i}}{|P_{i}|}\sum_{p\in P_{i}}\log\frac{\exp(\mathrm{sim}\left(f_{i},f_{p}\right)/\tau )}{\sum_{q\in N_{i}}{\left(\frac{1}{N_{c_{q}}}\right)}^{\gamma }\exp(\mathrm{sim}\left(f_{i},f_{q}/\tau \right)} \end{array}$$

where $P_{i}$ denotes the data belonging to the same fault type as $x_{i}$, $N_{i}$ denotes the data belonging to different fault types from $x_{i}$, and τ represents a temperature parameter that adjusts the smoothness of similarity measurement, thereby affecting the sensitivity of the loss function to the similarity between data samples; $N_{c_{q}}$ denotes the total number of samples in the class to which the negative sample *q* belongs; hyper-parameter γ modulate the intensity of class re-weighting.

#### 3.3.3. Distribution Alignment Loss

To achieve more accurate distribution alignment, this paper simultaneously considers global MMD-based distribution alignment and class distribution alignment implemented via weighted supervised contrastive learning in the distribution alignment component. The total distribution alignment loss is given as follows:

(10) $$\begin{array}{c} L_{align}=L_{mmd}+L_{w} \end{array}$$

### 3.4. Pseudo-Label Enhancement Module

In the pseudo-label enhancement module, the categories of source domain data can be explicitly determined, and the accurate prediction of source domain data is achieved by minimizing the cross-entropy loss. For target domain data, since the effectiveness of the domain alignment stage is affected by the quality of sample pseudo-labels, the reliability of target domain samples is improved by introducing pseudo-label enhancement for the target domain.

#### 3.4.1. Classifier Optimization

For the predicted labels of augmented source domain samples ${\hat{Y}}_{a}={\left\{{\hat{y}}_{a}\right\}}_{i=1}^{n_{s}}$, the predicted labels of original source domain samples $\hat{Y}={\left\{\hat{y}\right\}}_{i=1}^{n_{s}}$, the ground-truth labels of augmented source domain samples $Y_{a}={\left\{y_{a}\right\}}_{i=1}^{n_{s}}$, and the ground-truth labels of original source domain samples $Y={\left\{y\right\}}_{i=1}^{n_{s}}$, the corresponding cross-entropy loss $L_{c}$ is given as follows:

(11) $$\begin{array}{c} L_{c}=-\sum_{i=1}^{n_{s}}\sum_{c=1}^{C}\left[y_{a,c}\cdot log\left({\hat{y}}_{a,c}\right)+y_{c}\cdot log\left({\hat{y}}_{c}\right)\right] \end{array}$$

where *C* denotes the total number of classes.

#### 3.4.2. Pseudo-Label Enhancement

Hard samples are adaptively selected based on the results of sample classification difficulty scores. Fine-grained alignment within the target domain is achieved by constructing a Pseudo-Contrastive Matrix (PCM), which corrects erroneous pseudo-labels and enhances the compactness of feature clustering. For target domain samples $X_{all}^{t}=\left\{X^{t},X_{a}^{t}\right\}$, their corresponding classification difficulty-aware scores are denoted as $S(X_{all}^{t})$. According to the magnitude of the classification difficulty scores, target domain samples are filtered, and the top σ proportion of target domain samples are selected for pseudo-label enhancement. The predicted values corresponding to the filtered samples are denoted as $P={\left\{p\right\}}_{i=1}^{n_{\sigma }}$, which are further represented as $\hat{Y}={\left\{\hat{y}\right\}}_{i=1}^{n_{\sigma }}$ after one-hot encoding. A Pseudo-Label Matrix (PLM) is constructed, where ${\left(PLM\right)}_{i,j}={\hat{y}}_{i}^{T}\cdot {\hat{y}}_{j}$. The Pseudo-Contrastive Matrix (PCM) is constructed as follows:

(12) $$\begin{array}{c} {\left(PCM\right)}_{i,j}=\frac{exp(p_{i}\cdot p_{j}/t)}{\sum_{k=1}^{n_{\sigma }}exp\left(p_{i}\cdot p_{k}/t\right)} \end{array}$$

where t=0.15 is used to obtain the value of each element in the PCM. The loss function of pseudo-label enhancement is defined as the mean of the L1 distance between the PLM and the PCM, which aims to align the feature similarity between samples with the category relationship of pseudo-labels, thereby improving the confidence level of pseudo-labels in the target domain. The pseudo-label enhancement loss $L_{p}$ is given as follows:

(13) $$\begin{array}{c} L_{p}=mean(|PLM-PCM|) \end{array}$$

where PLM denotes the Pseudo-Label Matrix, and PCM denotes the Pseudo-Contrastive Matrix.

### 3.5. Total Model Loss

The total model loss consists of four components: the classification loss $L_{c}$, the pseudo-label enhancement loss $L_{p}$, the feature enhancement loss $L_{aug}$, and the alignment loss $L_{align}$. The total loss *L* is given as follows:

(14) $$\begin{array}{c} L=\lambda_{1}\cdot L_{c}+\lambda_{2}\cdot L_{p}+\lambda_{3}\cdot L_{aug}+\lambda_{4}\cdot L_{align} \end{array}$$

where $\lambda_{1}$, $\lambda_{2}$, $\lambda_{3}$, and $\lambda_{4}$ denote the weights of the respective losses in the total loss.

### 3.6. Algorithm Implementation Details

To better describe the algorithm proposed in this paper, the training process of the model is presented in Algorithm 1.
**Algorithm 1** Training Algorithm of ACLDA**Input:** 
Source domain samples $X^{s}$ and labels $Y^{s}$, target domain samples $X^{t}$; hyperparameter α, β, τ, $\lambda_{1}$, $\lambda_{2}$, $\lambda_{3}$ and $\lambda_{4}$; the total epoch number *N*, Augmentative difficulty cache (ADC) and Original difficulty cache (ODC).**Output:** 
The parameters $\theta_{f}$, $\theta_{f}$ of feature extractor *F* and classifier *C*. 1:set the values of $\alpha ,\beta ,\lambda_{1},\lambda_{2},\lambda_{3},\lambda_{4}$ and τ. 2:Randomly initialize $\theta_{f},\theta_{c}$ 3:Initialize ADC, ODC 4:**for** epoch∈{1,2,…,N} **do** 5:       $D_{s}=len(Dataloader\left(X^{s}\right)$ 6:       $D_{t}=len(Dataloader\left(X^{t}\right)$ 7:       $t=min(D_{s},D_{t})$ 8:       **for** batch∈1,2,…,t **do** 9:              $B_{a}^{s},Y_{a}^{s}=Augment\left(B^{s},Y^{s},ADC\right)$10:              $B_{a}^{t}=Augment\left(B^{t},ADC\right)$11:              $f^{s},f_{a}^{s},f^{t},f_{a}^{t}=F\left(B^{s},B_{a}^{s},B^{t},B_{a}^{t}\right)$12:              ${\hat{y}}^{s},{\hat{y}}_{a}^{s},{\hat{y}}^{t},{\hat{y}}_{a}^{t}=C\left(f^{s},f_{a}^{s},f^{t},f_{a}^{t}\right)$13:              loadandupdateADCandODCbyEquation(3)14:              $\mathrm{calculate}\;L_{aug}\;\mathrm{by}\;\mathrm{Equation}\;(6)$15:              $\mathrm{calculate}\;L_{align}\;\mathrm{by}\;\mathrm{Equation}\;(10)$16:              $\mathrm{calculate}\;L_{c}\;\mathrm{by}\;\mathrm{Equation}\;(11)$17:              $\mathrm{calculate}\;L_{p}\;\mathrm{by}\;\mathrm{Equation}\;(13)$18:              calculateLbyEquation(14)19:              $\mathrm{update}\theta_{f},\theta_{c}$20:       **end for**21:       $\mathrm{save}\;\mathrm{the}\;\mathrm{best}\;\mathrm{parameters}\;\theta_{f},\theta_{c}$22:**end for**

## 4. Experimental Evaluation

To verify the performance of the algorithm proposed in this paper, multiple unsupervised domain adaptation algorithms are evaluated on several datasets. The results of these algorithms are compared to demonstrate the superiority of the proposed algorithm. Meanwhile, to further validate the effectiveness of the improvements proposed in this paper, ablation experiments are designed to illustrate the contribution of each improvement to the performance enhancement of the algorithm.

### 4.1. Experimental Simulation Environment

The hardware environment for the experiments in this paper is as follows: a central processing unit (CPU) of Intel(R) Xeon(R) Platinum 8470Q, and a graphics card of NVIDIA RTX 5090 (32GB). The software environment includes: the Ubuntu 22.04 LTS operating system, the CUDA 12.8 computing acceleration toolkit, and the deep learning framework with Python 3.12 as the programming language and PyTorch 2.8.0 as the core library.

### 4.2. Data Description

As shown in Table 1, five datasets are selected as experimental datasets in this paper. Among them, four datasets (UCIHAR, HHAR, SSC and MFD) are used for comparative experiments, where the standard unsupervised domain adaptation benchmark framework ADATIME proposed in [34] is adopted to compare the proposed method with other competing methods. The CWRU dataset is selected as the ablation study dataset to analyze the impact of the proposed improvements on the model performance.

#### 4.2.1. UCIHAR Dataset

The UCIHAR dataset contains data from three types of sensors, namely accelerometers, gyroscopes, and body sensors, which were collected from 30 subjects. Each subject performed six activities: walking, walking upstairs, walking downstairs, standing, sitting, and lying down. Due to individual differences among the aforementioned subjects, each subject is regarded as an independent domain [35].

#### 4.2.2. HHAR Dataset

The Heterogeneous Human Activity Recognition (HHAR) dataset collects raw data from 9 subjects via sensor readings of smartphones and smartwatches. To reduce data heterogeneity, the sensor data of all selected subjects were obtained from the same model of smartphone. Each subject is regarded as an independent domain [36].

#### 4.2.3. SSC Dataset

The sleep stage classification task aims to divide electroencephalogram (EEG) signals into five sleep stages, namely wakefulness (W), non-rapid eye movement (NREM) sleep (N1, N2, N3), and rapid eye movement (REM) sleep [37]. This dataset contains electroencephalogram readings from 20 healthy subjects.

#### 4.2.4. MFD Dataset

The Mechanical Fault Diagnosis (MFD) dataset was collected by the University of Paderborn, which aims to identify various early faults through vibration signals. This dataset was acquired under four different operating conditions, and each condition is regarded as an independent domain in the experiments of this study [38].

#### 4.2.5. CWRU Dataset

The rolling bearing dataset is provided by Case Western Reserve University (CWRU). As a universally recognized benchmark dataset for evaluating and testing fault diagnosis methods, it includes four distinct health states: normal state, ball fault, inner race fault, and outer race fault. Each fault state is further subdivided into three different damage levels: 0.007 inches, 0.014 inches, and 0.021 inches. In addition, the experiments were conducted under four different load conditions, namely 0 horsepower, 1 horsepower, 2 horsepower, and 3 horsepower, with each load condition regarded as an independent domain [39].

### 4.3. Experimental Content

The experiments in this paper are divided into two parts: comparative experiments and ablation experiments. Comparative experiments compare the algorithm proposed in this paper with mainstream unsupervised domain adaptation algorithms to demonstrate the superiority of the proposed algorithm. To further verify the performance improvement of the proposed method on unsupervised domain adaptation algorithms, the ablation experiments compare the performance of a baseline model without the proposed modules with that of the algorithm integrated with the corresponding modules, thereby illustrating the impact of the proposed modules on unsupervised domain adaptation algorithms.

The comparative experiments adopted in this paper draw on the AdaTIME framework, where 12 unsupervised domain adaptation algorithms are implemented on four datasets for comparison with the proposed algorithm. To eliminate the impact of irrelevant variables on the results of comparative experiments, all algorithms in this experiment use the same feature extraction network and classifier. For each dataset, the hyperparameters of each algorithm are set to the optimal values for that dataset. Ten transfer scenarios are selected for each dataset to evaluate model performance. To address the impact of randomness in a single experiment on the credibility of results, each algorithm is run five times under each transfer scenario, and the average accuracy of the five runs is calculated to measure the performance of the algorithm on the corresponding transfer task for that dataset.

Ablation experiments are conducted on the CWRU dataset to analyze the role of key components in the model. These ablation experiments start with a baseline model without domain adaptation, which is obtained only by fine-tuning the feature extractor. In addition, MMDA is used as the second control model to verify the functions of the “Adaptive Data Augmentation (ADA) module” and the “Adaptive Weighted Contrastive Learning (AWCL) module” in the proposed algorithm. To verify the role of the ADA module in the model, the AWCL module is removed from the proposed model (w/o AWCL); to verify the role of the AWCL module, the ADA module is removed from the proposed model (w/o ADA). Since the proposed model is modified based on the MMDA model, the MMDA model can be regarded as the proposed method with both the ADA and AWCL modules removed (w/o AWCL + w/o ADA). Finally, the performance of the complete model is evaluated.

### 4.4. Analysis and Discussion of Comparative Experimental Results

The results of the comparative experiments are presented in Table 2, Table 3, Table 4 and Table 5, where each table shows the accuracy comparison between the algorithm proposed in this paper and other mainstream algorithms across 10 transfer scenarios on the corresponding dataset. It can be clearly observed from the data that, compared with other algorithms, the proposed algorithm does not show a significant advantage in accuracy on a single transfer task scenario. However, on average, the proposed algorithm achieves the best performance across all four datasets, indicating that it has better robustness. This benefit stems from the adaptive feature enhancement module proposed in this paper: on the one hand, adaptive data augmentation expands the number of dataset samples; on the other hand, it aligns data augmentation operations with the classification capability of the model. Through contrastive learning, the model can more easily extract important features of samples, which greatly improves feature quality. The combination of adaptive data augmentation and contrastive learning significantly enhances the generalization ability of the model.

The EEG dataset exhibits a significant class imbalance problem, where the number of minority class samples is much smaller than that of majority class samples. Compared with other datasets, the proposed algorithm achieves a more substantial performance improvement on the EEG dataset, demonstrating that it can still obtain favorable accuracy in class-imbalanced scenarios. This is attributed to the adaptive weighted contrastive learning module proposed in this paper. When the dataset is imbalanced, the model tends to perform poorly in classifying minority samples; the module assigns larger weights to minority samples, thereby mitigating the bias of model training toward majority samples caused by class imbalance.

### 4.5. Results and Discussion of Ablation Experiments

The results of the ablation experiments are presented in Table 6. When the adaptive feature enhancement module is applied independently, the model performance outperforms that of the baseline model. When the adaptive weighted contrastive learning module is applied alone, the model performance also exceeds that of the baseline model. When the adaptive data augmentation module and the adaptive weighted contrastive learning module work jointly, the model achieves better performance than either module applied individually, and its performance is significantly superior to that of the baseline model. This result further demonstrates that the proposed improvements are beneficial to the model performance.

**Figure 3:**
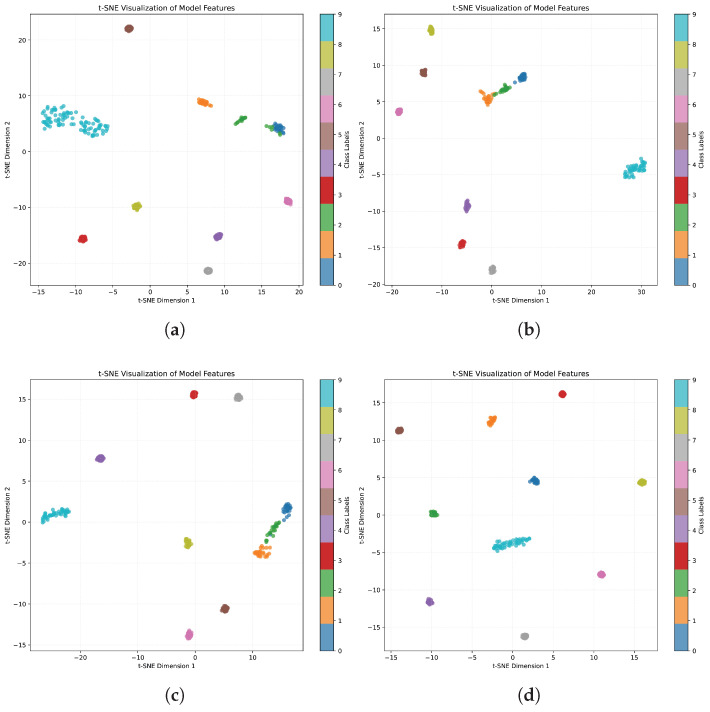
t-SNE Visualization of Ablation Study. (**a**) Description of baseline model. (**b**) Description of our model (w/o afa). (**c**) Description of our moedl (w/o wacl). (**d**) Description of our model.

To intuitively evaluate the contribution of each component in the proposed ACLDA framework, we visualize the learned feature representations using t-SNE embeddings on the CWRU dataset. Figure 3 illustrates the feature distributions under four different settings: (a) Description of baseline model, (b) Description of our model(w/o afa), (c) Description of our model(w/o wacl), and (d) Description of our model. In these plots, different colors represent different classes (or domains), where points from the target domains are projected into the same 2D space. As depicted in Figure 3d, the result demonstrates the effectiveness of the complete ACLDA framework. It is evident that the feature distributions of the source and target domains are well-aligned, showing a high degree of overlap. Moreover, the features exhibit clear cluster structures with high intra-class compactness and large inter-class separability. This superior visualization result confirms that the synergistic interaction between the difficulty perception mechanism and the weighted alignment strategy enables the model to learn highly discriminative representations, significantly boosting classification performance on the target domain.

Through the analysis of the ablation experiment results, it can be concluded that the feature enhancement module is beneficial for improving intra-class compactness and reducing domain discrepancies. The adaptive weighted contrastive learning module contributes to intra-class compactness and inter-class discriminability. By combining these two modules, the intra-class compactness is enhanced and the inter-class decision boundaries are more distinct, which is favorable for improving the classification performance of the model. Meanwhile, the reduction in the feature distribution discrepancy between the source and target domains indicates that the proposed model has stronger robustness.

### 4.6. Convergence and Stability Analysis

**Figure 4:**
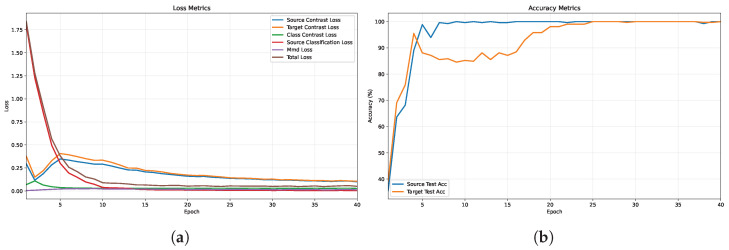
(**a**) Description of training loss curves. (**b**) Description of the test accuracy curves.

To validate the effectiveness and robustness of the proposed method, we monitored the descending trends of various loss functions and the accuracy variations throughout the training process, which spanned 40 epochs. Figure 4a,b illustrate training loss curves and the test accuracy curves, respectively.

The model exhibits high learning efficiency, with source accuracy saturating near 100% by epoch 10 and target accuracy stabilizing by epoch 25, indicating effective cross-domain knowledge transfer. This is corroborated by the loss curves, where the total loss drops sharply in the initial phase, driven by the classification loss, while contrastive losses decrease smoothly to optimize feature discriminability. Post-convergence behavior (after epoch 20) is characterized by minimal fluctuation in accuracy and consistently low MMD loss. This sustained stability through epoch 40 confirms the method’s resilience against overfitting and negative transfer.

### 4.7. Complexity and Efficiency Analysis

To assess the scalability and practical applicability of the proposed framework, we analyzed its computational complexity and runtime performance.

**Figure 5:**
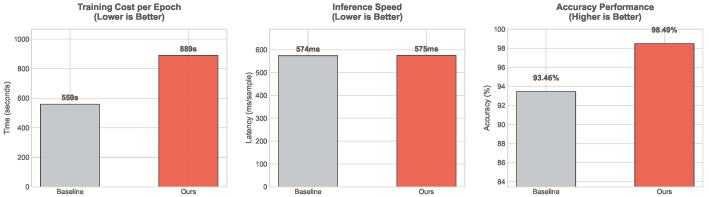
Comparison of training cost, inference latency, and model accuracy against the baseline.

Empirically, we compared the training time, inference latency, and accuracy of our method against the baseline on the target dataset. As shown in Figure 5, our method requires 889 s per epoch for training, compared to 559 s for the baseline. This increase is primarily attributed to the additional gradient computations required for the contrastive loss. However, this training overhead is a worthwhile trade-off for the significant performance gain, with accuracy improving from 93.46% to 98.49%. Crucially, for real-world deployment, the inference speed remains virtually unaffected. Since the difficulty-aware weighting and augmentation modules are training-time regularization techniques, they are detached during inference. Consequently, our model achieves a total inference time of 575 ms for the entire test set, comparable to the baseline’s 574 ms. This demonstrates that our method enhances generalization without compromising real-time processing efficiency.

### 4.8. Sensitivity Analysis on Augmentation Magnitude

**Figure 6:**
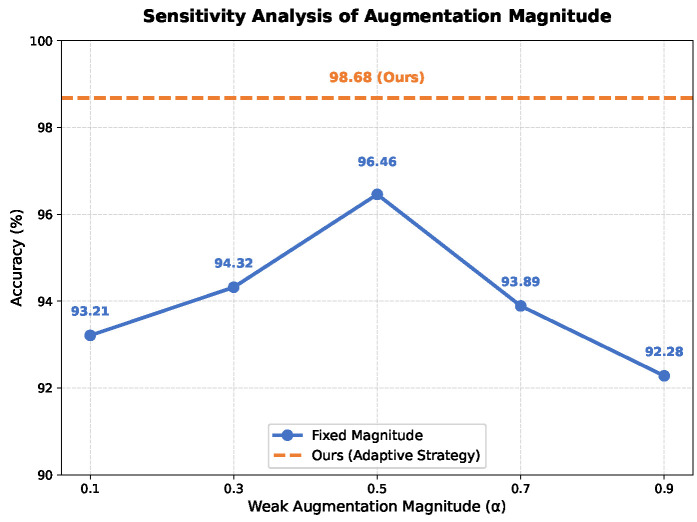
Sensitivity Analysis of Augmentation Magnitude.

As shown in Figure 6, when employing fixed data augmentation strategies, weak augmentation fails to guarantee sample diversity, while overly strong augmentation compromises sample semantics. Both scenarios lead to performance degradation. To address this, the adaptive data augmentation method proposed in this paper effectively operates within a “safe” semantic boundary.

### 4.9. Comparison of Difficulty Estimation Metrics

**Figure 7:**
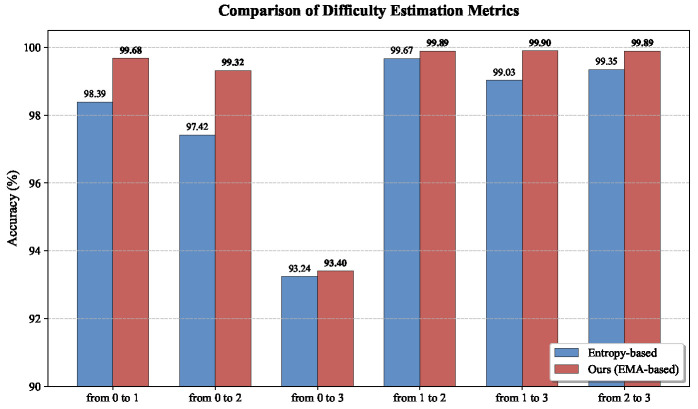
Comparison of Different Difficulty Estimation Metrics.

To address the concern about the stability of our difficulty estimation mechanism, we have conducted comparative experiments with the standard Entropy-based difficulty metric. As shown in Figure 7, our EMA-based method consistently outperforms the Entropy-based baseline in all scenarios.

## 5. Conclusions

In this paper, we integrate the unsupervised domain adaptation (UDA) algorithm with contrastive learning (CL), and propose a novel unsupervised domain adaptation algorithm based on adaptive contrastive learning. Different from existing methods, our contributions are twofold: first, we introduce an adaptive intra-domain contrastive learning strategy, which enables the model to perform adaptive data augmentation on raw samples according to its own learning capability, and then leverages both augmented and raw samples for contrastive learning, thereby enhancing the quality of features extracted by the model. Second, unlike traditional distribution alignment schemes, we adopt adaptive weighted supervised contrastive learning for class-level alignment; this method allows the model to focus on samples that are conducive to improving its prediction accuracy, while also facilitating the enlargement of inter-class differences and the enhancement of intra-class compactness. In the comparative experiments, the proposed algorithm achieves state-of-the-art performance on four benchmark datasets, which verifies its superiority and favorable robustness. Moreover, the results of ablation experiments demonstrate that the proposed method contributes to the performance improvement of domain adaptation algorithms, confirming its practical value.

## Figures and Tables

**Table 1:** Dataset Description.

Dataset	Domains	Channels	Classes	Sequence Length	Training	Testing
UCIHAR	30	9	6	128	2300	990
HHAR	9	3	6	128	12,716	5218
SSC	20	1	5	3000	14,280	6130
MFD	4	1	3	5120	7312	3604
CWRU	4	1	10	1200	1228	311

**Table 2:** PerformanceComparison on the HAR Dataset.

Transfer	Deep_Coral	MMDA	DANN	CDAN	DIRT	DSAN	HoMM	DDC	CoDATS	AdvSKM	SASA	CoTMix	Ours
12_to_16	0.7152	0.7000	0.6909	0.6909	0.6576	0.7364	0.7515	0.6970	0.7273	0.7303	0.6818	0.7000	**0.7925**
18_to_27	1.0000	1.0000	1.0000	1.0000	0.9794	1.0000	1.0000	1.0000	1.0000	1.0000	0.9794	1.0000	**1.0000**
20_to_5	0.8681	0.8864	0.8608	0.8608	**0.9011**	0.8498	0.8681	0.8535	0.8608	0.8352	0.8388	0.8132	0.8835
24_to_8	0.9490	0.9647	0.9647	0.9451	**0.9882**	0.9686	0.9647	0.9020	0.9294	0.8980	0.9176	0.8627	0.9295
28_to_27	0.8083	0.7876	0.8289	0.8643	0.9174	**0.9705**	0.8289	0.7906	0.8643	0.8083	0.9292	0.9646	0.9559
2_to_11	1.0000	1.0000	1.0000	1.0000	0.9860	1.0000	1.0000	1.0000	0.9544	1.0000	1.0000	0.9544	**1.0000**
30_to_20	0.8037	0.8162	0.7570	**0.8536**	0.8349	0.8318	0.8006	0.7850	0.8006	0.7850	0.6854	0.7726	0.8173
6_to_23	0.9643	0.9643	0.9762	0.9643	0.9643	**1.0000**	0.9643	0.9583	0.9643	0.9494	0.9554	0.9643	0.9616
7_to_13	0.9293	0.9394	0.9327	0.9394	0.9394	0.9394	0.9327	0.9259	0.9293	0.9293	0.9293	0.9293	**0.9600**
9_to_18	0.7121	0.7879	0.8242	0.8030	**0.9394**	0.8515	0.7970	0.6848	0.8333	0.7121	0.6394	0.8606	0.8737
Average	0.8750	0.8847	0.8835	0.8921	0.9108	0.9148	0.8908	0.8597	0.8864	0.8648	0.8556	0.8822	**0.9174**

The bold values indicate the best performance for each transfer task.

**Table 3:** Performance Comparison on the EEG Dataset.

Transfer	Deep_Coral	MMDA	DANN	CDAN	DIRT	DSAN	HoMM	DDC	CoDATS	AdvSKM	SASA	CoTMix	Ours
0_to_11	0.5530	0.5074	0.5048	0.4534	0.3597	0.4258	0.5543	0.6114	0.4624	0.5986	0.4393	0.5851	**0.6210**
12_to_5	0.6991	0.7824	0.7350	0.7448	0.7037	**0.7894**	0.6869	0.6823	0.6348	0.7002	0.7159	0.7182	0.7295
13_to_17	0.6507	**0.7383**	0.5830	0.6251	0.5314	0.5550	0.6158	0.6563	0.5470	0.6251	0.5706	0.7231	0.7060
16_to_1	0.6972	0.7123	0.6924	0.7230	**0.7933**	0.7074	0.6308	0.6744	0.7026	0.7089	0.6769	0.5851	0.7835
18_to_12	0.6147	0.6349	0.6289	0.6262	0.5211	0.4866	0.6196	0.6388	0.5298	0.5900	0.5534	0.5495	**0.6720**
3_to_19	0.7367	**0.8339**	0.7228	0.7849	0.7679	0.7189	0.6999	0.6868	0.7204	0.7129	0.6900	0.7335	0.8310
5_to_15	0.7935	**0.8859**	0.8245	0.8740	0.7673	0.8213	0.8160	0.8344	0.8074	0.8462	0.6699	0.7264	0.8305
6_to_2	0.7660	0.7600	0.7572	0.7643	0.6465	0.7572	0.7665	0.7561	0.7474	0.7540	0.7398	0.6841	**0.8015**
7_to_18	0.7456	0.7756	0.7633	0.7691	0.7126	0.7768	0.7091	0.7232	0.7403	0.7397	0.7002	**0.8009**	0.7750
9_to_14	0.7819	0.7058	0.7922	0.8279	0.8507	0.8052	0.7785	0.7973	0.8302	0.8217	0.8268	0.7513	**0.8560**
Average	0.7038	0.7337	0.7004	0.7193	0.6654	0.6844	0.6877	0.7061	0.6722	0.7097	0.6583	0.6857	**0.7606**

The bold values indicate the best performance for each transfer task.

**Table 4:** Performance Comparison on the HHAR Dataset.

Transfer	Deep_Coral	MMDA	DANN	CDAN	DIRT	DSAN	HoMM	DDC	CoDATS	AdvSKM	SASA	CoTMix	Ours
0_to_2	0.7565	0.7614	0.7853	**0.8140**	0.8091	0.6751	0.7074	0.6456	0.7544	0.6786	0.7726	0.7404	0.7549
0_to_6	0.6753	0.5489	0.5256	0.4664	0.5469	0.5376	0.6547	0.6547	0.4478	0.5862	0.5442	0.6727	**0.6891**
1_to_6	0.8589	0.9082	**0.9381**	0.9308	0.9381	0.9301	0.8889	0.7126	0.9148	0.7126	0.9128	0.9168	0.9116
2_to_7	0.4642	0.4795	0.5087	0.5094	0.6486	0.5331	0.4551	0.4976	0.4022	0.4356	0.4537	**0.6743**	0.5123
3_to_8	0.7934	0.9454	0.8681	0.8083	0.9688	**0.9805**	0.8096	0.7843	0.9311	0.7940	0.8493	0.8272	0.8107
4_to_5	0.8788	0.9349	0.9587	**0.9774**	0.9429	0.9768	0.9284	0.8369	0.7995	0.8214	0.9368	0.7202	0.9188
5_to_0	0.3705	0.4376	0.3377	0.3516	0.2159	0.2961	0.3450	0.3428	0.3406	0.3508	0.2925	**0.6441**	0.6091
6_to_1	0.8545	0.9092	0.9248	0.9229	**0.9726**	0.9447	0.8532	0.7494	0.9011	0.8041	0.9098	0.8744	0.9311
7_to_4	0.8257	0.8743	0.9528	0.9574	**0.9734**	0.9474	0.8896	0.8230	0.9235	0.8323	0.9042	0.9148	0.9139
8_to_3	0.8972	0.8461	0.9657	0.9716	0.9519	**0.9730**	0.9664	0.7520	0.8082	0.7987	0.9599	0.8775	0.9586
Average	0.7375	0.7645	0.7765	0.7710	0.7968	0.7794	0.7498	0.6799	0.7223	0.6814	0.7536	0.7862	**0.8010**

The bold values indicate the best performance for each transfer task.

**Table 5:** Performance Comparison on the FD Dataset.

Transfer	Deep_Coral	MMDA	DANN	CDAN	DIRT	DSAN	HoMM	DDC	CoDATS	AdvSKM	SASA	CoTMix	Ours
0_to_1	0.7425	0.8835	0.8172	0.8431	0.8202	0.8276	0.7680	0.7629	0.7063	0.7484	0.7029	0.5953	**0.8035**
0_to_3	0.7532	**0.9434**	0.8350	0.8391	0.8420	0.8228	0.7695	0.7932	0.8598	0.7529	0.7555	0.6226	0.7645
1_to_0	0.8513	0.8594	0.8790	0.9016	0.8061	0.8010	0.8424	0.8061	0.8935	0.8502	0.8446	0.8047	**0.9630**
1_to_2	0.8176	**0.9267**	0.8117	0.8257	0.9034	0.8461	0.8135	0.8376	0.9031	0.8091	0.8309	0.7962	0.8710
1_to_3	0.9682	0.9812	0.9896	1.0000	1.0000	0.9996	0.9996	0.9996	0.9993	0.9982	0.9837	0.8442	**1.0000**
2_to_1	0.9837	0.9796	0.9575	**0.9989**	0.9904	0.9353	0.9808	0.9094	0.9334	0.9541	0.9885	0.7784	0.9695
2_to_3	0.9863	1.0000	0.9671	**1.0000**	0.9885	0.9589	0.9837	0.9279	0.9871	0.9478	0.9131	0.7573	0.9980
3_to_0	0.8280	0.8290	0.8576	0.8391	0.7769	0.7876	0.8361	0.8065	0.8890	0.8313	0.8417	0.8472	**0.9620**
3_to_1	1.0000	0.9653	0.9996	1.0000	1.0000	0.9989	1.0000	0.9800	0.9830	0.9982	0.9982	0.8387	**1.0000**
3_to_2	0.8080	**0.9375**	0.8106	0.8121	0.9034	0.8898	0.8147	0.8191	0.8953	0.7873	0.8143	0.7913	0.9140
Average	0.8739	0.9306	0.8925	0.9060	0.9031	0.8868	0.8808	0.8642	0.9050	0.8677	0.8673	0.7676	**0.9346**

The bold values indicate the best performance for each transfer task.

**Table 6:** Ablation study using CWRU dataset.

Methods	$T_{0\to 1}$	$T_{0\to 2}$	$T_{0\to 3}$	$T_{1\to 2}$	$T_{1\to 3}$	$T_{2\to 3}$	Average
No Adaptation	86.59±0.97	84.24±0.32	84.45±0.18	97.20±2.14	92.17±0.18	98.28±0.98	90.49
Our (w/o afa + w/o wacl)	90.88±1.93	88.41±0.85	84.77±0.18	98.49±1.34	98.81±0.66	99.38±0.79	93.46
Our (w/o afa)	97.75±2.12	91.44±1.13	89.42±2.20	98.89±1.05	99.60±0.30	99.32±0.31	96.06
Our (w/o wacl)	98.18±1.2	93.60±2.10	91.01±1.12	99.68±0.28	99.56±0.32	99.55±0.45	96.93
Our	99.68±0.32	99.32±0.56	93.40±0.78	99.89±0.04	99.90±0.09	99.89±0.10	98.68

The bold values indicate the best performance for each transfer task.

## Data Availability

The original data presented in the study are openly available on GitHub at https://github.com/emadeldeen24/AdaTime?tab=readme-ov-file (accessed on 3 February 2026).
